# Crystal structure of 1,1,2,2-tetra­methyl-1,2-bis­(2,3,4,5-tetra­methyl­cyclo­penta-2,4-dien-1-yl)disilane

**DOI:** 10.1107/S2056989015019891

**Published:** 2015-10-28

**Authors:** Christian Godemann, Anke Spannenberg, Torsten Beweries

**Affiliations:** a Leibniz-Institut für Katalyse e. V. der Universität Rostock, Albert-Einstein-Strasse 29a, 18059 Rostock, Germany

**Keywords:** crystal structure, disilane, ansa ligand

## Abstract

The mol­ecular structure of the title compound, C_22_H_38_Si_2_, features a *trans* arrangement of the cyclo­penta­dienyl rings to avoid steric strain [C—Si—Si—C torsion angle = −179.0 (5)°]. The Si—Si bond length is 2.3444 (4) Å. The most notable inter­molecular inter­actions in the mol­ecular packing are C—H⋯π contacts that lead to the formation of wave-like supra­molecular chains along the *b* axis.

## Related literature   

For synthesis of the title compound, see: Kessler *et al.* (2013 ▸). For group 4 complexes with this ligand, see: Godemann *et al.* (2014 ▸, 2015 ▸); Pinkas *et al.* (2011 ▸); Xu *et al.* (1997 ▸); Horáček *et al.* (2008 ▸).
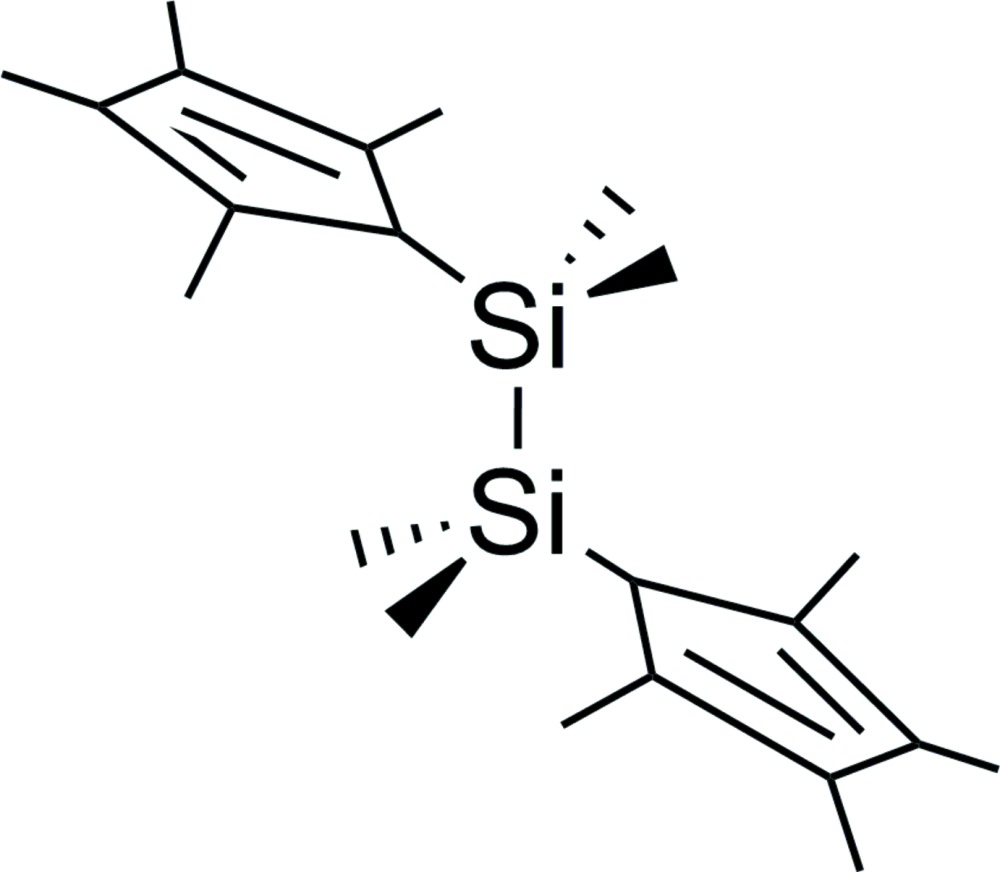


## Experimental   

### Crystal data  

C_22_H_38_Si_2_
*M* *_r_* = 358.70
Monoclinic 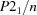

*a* = 8.7790 (2) Å
*b* = 15.3039 (4) Å
*c* = 16.4355 (4) Å
β = 93.678 (1)°
*V* = 2203.61 (9) Å^3^
*Z* = 4
Mo *K*α radiation
μ = 0.16 mm^−1^
*T* = 150 K
0.55 × 0.41 × 0.29 mm

### Data collection  

Bruker APEXII CCD diffractometer
Absorption correction: multi-scan (*SADABS*; Bruker, 2008 ▸) *T*_min_ = 0.92, *T*_max_ = 0.95
46817 measured reflections
5318 independent reflections
4636 reflections with *I* > 2σ(*I*)
*R*_int_ = 0.035

### Refinement  

*R*[*F* ^2^ > 2σ(*F* ^2^)] = 0.034
*wR*(*F* ^2^) = 0.097
*S* = 1.06
5318 reflections
229 parameters
H-atom parameters constrained
Δρ_max_ = 0.32 e Å^−3^
Δρ_min_ = −0.23 e Å^−3^

### 

Data collection: *APEX2* (Bruker, 2011 ▸); cell refinement: *SAINT* (Bruker, 2009 ▸); data reduction: *SAINT*; program(s) used to solve structure: *SHELXS97* (Sheldrick, 2008 ▸); program(s) used to refine structure: *SHELXL97* (Sheldrick, 2008 ▸); molecular graphics: *XP* in *SHELXTL* (Sheldrick, 2008 ▸); software used to prepare material for publication: *SHELXL97*.

## Supplementary Material

Crystal structure: contains datablock(s) I, New_Global_Publ_Block. DOI: 10.1107/S2056989015019891/tk5400sup1.cif

Structure factors: contains datablock(s) I. DOI: 10.1107/S2056989015019891/tk5400Isup2.hkl

Supporting information file. DOI: 10.1107/S2056989015019891/tk5400Isup3.cml

**Fig. 1.**
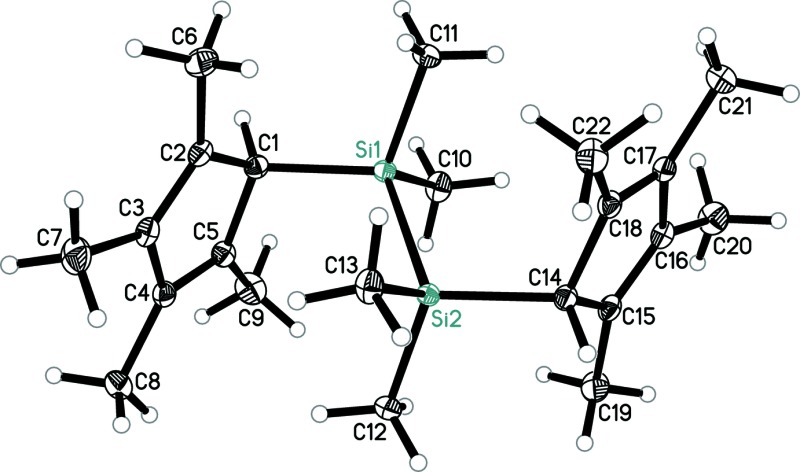
Molecular structure of the title compound with atom labelling scheme and displacement ellipsoids drawn at 30% probability level.

. DOI: 10.1107/S2056989015019891/tk5400fig1.tif
Mol­ecular structure of the title compound with atom labelling scheme and displacement ellipsoids drawn at 30% probability level.

CCDC reference: 1432476

Additional supporting information:  crystallographic information; 3D view; checkCIF report

## Figures and Tables

**Table 1:** Hydrogen-bond geometry (, ) *Cg*1 is the centroid of the C14C18 ring.

*D*H*A*	*D*H	H*A*	*D* *A*	*D*H*A*
C1H1*Cg*1^i^	1.00	2.76	3.7350(13)	166

Symmetry code: (i) 
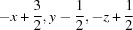
.

## supplementary crystallographic information

### S1. Synthesis and crystallization

The synthesis of the title compound has been described previously (Kessler *et al.*, 2013). A saturated solution of the title compound in *n*-hexane was very slowly cooled from 60 °C to room temperature resulting in precipitation of colourless crystals.

### S2. Refinement

H atoms were placed in idealized positions with d(C—H) = 1.00 Å (CH) & 0.98 Å (CH_3_), and refined using a riding model with *U*_iso_(H) fixed at 1.2 *U*_eq_(C) for CH & 1.5 *U*_eq_(C) for CH_3_.

### Crystal data

**Table:**

C_22_H_38_Si_2_	*F*(000) = 792
*M**_r_* = 358.70	*D*_x_ = 1.081 Mg m^−^^3^
Monoclinic, *P*2_1_/*n*	Mo *K*α radiation, λ = 0.71073 Å
*a* = 8.7790 (2) Å	Cell parameters from 9858 reflections
*b* = 15.3039 (4) Å	θ = 2.5–28.6°
*c* = 16.4355 (4) Å	µ = 0.16 mm^−^^1^
β = 93.678 (1)°	*T* = 150 K
*V* = 2203.61 (9) Å^3^	Prism, colourless
*Z* = 4	0.55 × 0.41 × 0.29 mm

### Data collection

**Table:**

Bruker APEXII CCD diffractometer	5318 independent reflections
Radiation source: fine-focus sealed tube	4636 reflections with *I* > 2σ(*I*)
Curved graphite monochromator	*R*_int_ = 0.035
Detector resolution: 8.3333 pixels mm^-1^	θ_max_ = 28.0°, θ_min_ = 1.8°
φ and ω scans	*h* = −11→11
Absorption correction: multi-scan (*SADABS*; Bruker, 2008)	*k* = −20→19
*T*_min_ = 0.92, *T*_max_ = 0.95	*l* = −21→21
46817 measured reflections	

### Refinement

**Table:**

Refinement on *F*^2^	Primary atom site location: structure-invariant direct methods
Least-squares matrix: full	Secondary atom site location: difference Fourier map
*R*[*F*^2^ > 2σ(*F*^2^)] = 0.034	Hydrogen site location: inferred from neighbouring sites
*wR*(*F*^2^) = 0.097	H-atom parameters constrained
*S* = 1.06	*w* = 1/[σ^2^(*F*_o_^2^) + (0.047*P*)^2^ + 0.8183*P*] where *P* = (*F*_o_^2^ + 2*F*_c_^2^)/3
5318 reflections	(Δ/σ)_max_ = 0.001
229 parameters	Δρ_max_ = 0.32 e Å^−^^3^
0 restraints	Δρ_min_ = −0.23 e Å^−^^3^

### Special details

**Table:**

Geometry. All e.s.d.'s (except the e.s.d. in the dihedral angle between two l.s. planes) are estimated using the full covariance matrix. The cell e.s.d.'s are taken into account individually in the estimation of e.s.d.'s in distances, angles and torsion angles; correlations between e.s.d.'s in cell parameters are only used when they are defined by crystal symmetry. An approximate (isotropic) treatment of cell e.s.d.'s is used for estimating e.s.d.'s involving l.s. planes.
Refinement. Refinement of *F*^2^ against ALL reflections. The weighted *R*-factor *wR* and goodness of fit *S* are based on *F*^2^, conventional *R*-factors *R* are based on *F*, with *F* set to zero for negative *F*^2^. The threshold expression of *F*^2^ > σ(*F*^2^) is used only for calculating *R*-factors(gt) *etc*. and is not relevant to the choice of reflections for refinement. *R*-factors based on *F*^2^ are statistically about twice as large as those based on *F*, and *R*- factors based on ALL data will be even larger.

### Fractional atomic coordinates and isotropic or equivalent isotropic displacement parameters (Å^2^)

**Table:**

	*x*	*y*	*z*	*U*_iso_*/*U*_eq_	
C1	0.85658 (13)	0.71127 (8)	0.10459 (7)	0.0212 (2)	
H1	0.8437	0.6501	0.1244	0.025*	
C2	1.01814 (13)	0.72631 (8)	0.08367 (8)	0.0227 (2)	
C3	1.01837 (14)	0.75224 (8)	0.00516 (8)	0.0243 (2)	
C4	0.86221 (14)	0.75132 (8)	−0.03149 (7)	0.0234 (2)	
C5	0.76715 (14)	0.72439 (8)	0.02488 (7)	0.0228 (2)	
C6	1.15522 (16)	0.71322 (10)	0.14202 (9)	0.0349 (3)	
H6A	1.2403	0.6908	0.1123	0.052*	
H6B	1.1304	0.6712	0.1842	0.052*	
H6C	1.1843	0.7691	0.1676	0.052*	
C7	1.15333 (17)	0.77834 (11)	−0.04066 (10)	0.0374 (3)	
H7A	1.2463	0.7744	−0.0045	0.056*	
H7B	1.1400	0.8385	−0.0601	0.056*	
H7C	1.1620	0.7392	−0.0873	0.056*	
C8	0.81945 (17)	0.77582 (10)	−0.11828 (8)	0.0332 (3)	
H8A	0.8533	0.7299	−0.1546	0.050*	
H8B	0.8687	0.8312	−0.1311	0.050*	
H8C	0.7084	0.7823	−0.1259	0.050*	
C9	0.59858 (16)	0.70960 (11)	0.01139 (9)	0.0359 (3)	
H9A	0.5448	0.7653	0.0159	0.054*	
H9B	0.5648	0.6688	0.0525	0.054*	
H9C	0.5760	0.6850	−0.0431	0.054*	
C10	0.59182 (15)	0.77168 (9)	0.20801 (9)	0.0298 (3)	
H10A	0.5796	0.7097	0.2212	0.045*	
H10B	0.5237	0.7867	0.1605	0.045*	
H10C	0.5661	0.8075	0.2546	0.045*	
C11	0.91263 (16)	0.77522 (9)	0.28252 (8)	0.0283 (3)	
H11A	0.8710	0.8103	0.3258	0.042*	
H11B	1.0184	0.7928	0.2756	0.042*	
H11C	0.9099	0.7132	0.2973	0.042*	
C12	0.69980 (16)	0.95635 (9)	0.04136 (7)	0.0275 (3)	
H12A	0.6950	1.0192	0.0299	0.041*	
H12B	0.5966	0.9340	0.0475	0.041*	
H12C	0.7448	0.9260	−0.0038	0.041*	
C13	1.02293 (15)	0.96094 (9)	0.11437 (8)	0.0281 (3)	
H13A	1.0465	0.9302	0.0644	0.042*	
H13B	1.0925	0.9412	0.1597	0.042*	
H13C	1.0353	1.0240	0.1066	0.042*	
C14	0.76149 (13)	1.01788 (8)	0.21979 (7)	0.0205 (2)	
H14	0.7769	1.0788	0.1999	0.025*	
C15	0.59856 (13)	1.00765 (8)	0.24095 (7)	0.0223 (2)	
C16	0.59561 (14)	0.98747 (8)	0.32073 (8)	0.0236 (2)	
C17	0.75209 (14)	0.98656 (8)	0.35759 (7)	0.0228 (2)	
C18	0.84985 (13)	1.00645 (8)	0.30048 (7)	0.0222 (2)	
C19	0.46292 (15)	1.01960 (10)	0.18157 (9)	0.0334 (3)	
H19A	0.4372	0.9637	0.1550	0.050*	
H19B	0.4872	1.0627	0.1403	0.050*	
H19C	0.3758	1.0402	0.2107	0.050*	
C20	0.45939 (16)	0.97111 (10)	0.36880 (10)	0.0367 (3)	
H20A	0.4494	1.0185	0.4082	0.055*	
H20B	0.4722	0.9155	0.3979	0.055*	
H20C	0.3674	0.9686	0.3318	0.055*	
C21	0.78832 (18)	0.96916 (10)	0.44647 (8)	0.0346 (3)	
H21A	0.8990	0.9635	0.4570	0.052*	
H21B	0.7385	0.9149	0.4620	0.052*	
H21C	0.7511	1.0177	0.4786	0.052*	
C22	1.01980 (15)	1.01596 (10)	0.31332 (9)	0.0324 (3)	
H22A	1.0463	1.0338	0.3697	0.049*	
H22B	1.0554	1.0604	0.2760	0.049*	
H22C	1.0687	0.9599	0.3026	0.049*	
Si1	0.79503 (4)	0.79312 (2)	0.184414 (19)	0.01845 (9)	
Si2	0.82065 (4)	0.93696 (2)	0.138159 (18)	0.01799 (9)	

### Atomic displacement parameters (Å^2^)

**Table:**

	*U*^11^	*U*^22^	*U*^33^	*U*^12^	*U*^13^	*U*^23^
C1	0.0224 (6)	0.0182 (5)	0.0231 (6)	−0.0005 (4)	0.0010 (4)	0.0000 (4)
C2	0.0193 (6)	0.0207 (5)	0.0281 (6)	0.0035 (4)	0.0009 (5)	−0.0026 (5)
C3	0.0220 (6)	0.0228 (6)	0.0287 (6)	0.0019 (4)	0.0058 (5)	−0.0031 (5)
C4	0.0253 (6)	0.0226 (6)	0.0223 (6)	0.0024 (5)	0.0009 (5)	−0.0038 (5)
C5	0.0219 (6)	0.0223 (6)	0.0239 (6)	−0.0014 (4)	−0.0015 (4)	−0.0046 (5)
C6	0.0237 (6)	0.0428 (8)	0.0373 (7)	0.0086 (6)	−0.0046 (5)	−0.0002 (6)
C7	0.0292 (7)	0.0442 (8)	0.0402 (8)	0.0019 (6)	0.0139 (6)	0.0004 (6)
C8	0.0373 (7)	0.0390 (8)	0.0231 (6)	0.0037 (6)	0.0014 (5)	−0.0008 (5)
C9	0.0250 (7)	0.0465 (8)	0.0354 (7)	−0.0099 (6)	−0.0042 (5)	−0.0049 (6)
C10	0.0259 (6)	0.0287 (7)	0.0359 (7)	−0.0050 (5)	0.0114 (5)	−0.0005 (5)
C11	0.0353 (7)	0.0288 (6)	0.0204 (6)	0.0030 (5)	−0.0022 (5)	0.0033 (5)
C12	0.0346 (7)	0.0277 (6)	0.0200 (6)	0.0040 (5)	−0.0011 (5)	0.0023 (5)
C13	0.0264 (6)	0.0264 (6)	0.0328 (7)	−0.0051 (5)	0.0113 (5)	−0.0022 (5)
C14	0.0195 (5)	0.0203 (5)	0.0215 (5)	0.0016 (4)	0.0004 (4)	−0.0023 (4)
C15	0.0176 (5)	0.0216 (6)	0.0273 (6)	0.0035 (4)	−0.0006 (4)	−0.0049 (5)
C16	0.0217 (6)	0.0203 (5)	0.0290 (6)	0.0012 (4)	0.0040 (5)	−0.0033 (5)
C17	0.0260 (6)	0.0199 (5)	0.0222 (6)	0.0033 (4)	−0.0003 (5)	−0.0028 (4)
C18	0.0201 (6)	0.0215 (5)	0.0244 (6)	0.0021 (4)	−0.0024 (4)	−0.0060 (5)
C19	0.0222 (6)	0.0429 (8)	0.0342 (7)	0.0080 (6)	−0.0059 (5)	−0.0073 (6)
C20	0.0292 (7)	0.0395 (8)	0.0427 (8)	−0.0005 (6)	0.0126 (6)	0.0018 (6)
C21	0.0422 (8)	0.0362 (7)	0.0249 (6)	0.0042 (6)	−0.0009 (6)	0.0012 (6)
C22	0.0205 (6)	0.0409 (8)	0.0351 (7)	0.0000 (5)	−0.0042 (5)	−0.0099 (6)
Si1	0.01894 (16)	0.01846 (16)	0.01803 (16)	−0.00115 (11)	0.00177 (11)	0.00206 (11)
Si2	0.01903 (16)	0.01773 (16)	0.01736 (15)	−0.00037 (11)	0.00222 (11)	0.00092 (11)

### Geometric parameters (Å, º)

**Table:**

C1—C5	1.4969 (16)	C12—H12A	0.9800
C1—C2	1.4985 (16)	C12—H12B	0.9800
C1—Si1	1.9166 (12)	C12—H12C	0.9800
C1—H1	1.0000	C13—Si2	1.8789 (13)
C2—C3	1.3501 (18)	C13—H13A	0.9800
C2—C6	1.5033 (17)	C13—H13B	0.9800
C3—C4	1.4617 (17)	C13—H13C	0.9800
C3—C7	1.4984 (18)	C14—C15	1.5017 (16)
C4—C5	1.3505 (17)	C14—C18	1.5031 (16)
C4—C8	1.4993 (17)	C14—Si2	1.9216 (12)
C5—C9	1.4994 (17)	C14—H14	1.0000
C6—H6A	0.9800	C15—C16	1.3490 (17)
C6—H6B	0.9800	C15—C19	1.5016 (17)
C6—H6C	0.9800	C16—C17	1.4655 (17)
C7—H7A	0.9800	C16—C20	1.4961 (17)
C7—H7B	0.9800	C17—C18	1.3467 (17)
C7—H7C	0.9800	C17—C21	1.4989 (17)
C8—H8A	0.9800	C18—C22	1.5004 (17)
C8—H8B	0.9800	C19—H19A	0.9800
C8—H8C	0.9800	C19—H19B	0.9800
C9—H9A	0.9800	C19—H19C	0.9800
C9—H9B	0.9800	C20—H20A	0.9800
C9—H9C	0.9800	C20—H20B	0.9800
C10—Si1	1.8787 (13)	C20—H20C	0.9800
C10—H10A	0.9800	C21—H21A	0.9800
C10—H10B	0.9800	C21—H21B	0.9800
C10—H10C	0.9800	C21—H21C	0.9800
C11—Si1	1.8780 (13)	C22—H22A	0.9800
C11—H11A	0.9800	C22—H22B	0.9800
C11—H11B	0.9800	C22—H22C	0.9800
C11—H11C	0.9800	Si1—Si2	2.3444 (4)
C12—Si2	1.8783 (13)		
			
C5—C1—C2	103.28 (10)	Si2—C13—H13A	109.5
C5—C1—Si1	110.87 (8)	Si2—C13—H13B	109.5
C2—C1—Si1	111.67 (8)	H13A—C13—H13B	109.5
C5—C1—H1	110.3	Si2—C13—H13C	109.5
C2—C1—H1	110.3	H13A—C13—H13C	109.5
Si1—C1—H1	110.3	H13B—C13—H13C	109.5
C3—C2—C1	108.89 (11)	C15—C14—C18	103.23 (10)
C3—C2—C6	126.77 (12)	C15—C14—Si2	113.49 (8)
C1—C2—C6	124.33 (11)	C18—C14—Si2	113.19 (8)
C2—C3—C4	109.40 (11)	C15—C14—H14	108.9
C2—C3—C7	127.48 (12)	C18—C14—H14	108.9
C4—C3—C7	123.12 (12)	Si2—C14—H14	108.9
C5—C4—C3	108.95 (11)	C16—C15—C19	126.57 (12)
C5—C4—C8	126.95 (12)	C16—C15—C14	109.08 (10)
C3—C4—C8	124.10 (12)	C19—C15—C14	124.35 (11)
C4—C5—C1	109.19 (11)	C15—C16—C17	109.15 (11)
C4—C5—C9	126.30 (12)	C15—C16—C20	128.13 (12)
C1—C5—C9	124.51 (11)	C17—C16—C20	122.68 (12)
C2—C6—H6A	109.5	C18—C17—C16	109.40 (11)
C2—C6—H6B	109.5	C18—C17—C21	127.92 (12)
H6A—C6—H6B	109.5	C16—C17—C21	122.63 (11)
C2—C6—H6C	109.5	C17—C18—C22	126.61 (12)
H6A—C6—H6C	109.5	C17—C18—C14	108.99 (10)
H6B—C6—H6C	109.5	C22—C18—C14	124.40 (11)
C3—C7—H7A	109.5	C15—C19—H19A	109.5
C3—C7—H7B	109.5	C15—C19—H19B	109.5
H7A—C7—H7B	109.5	H19A—C19—H19B	109.5
C3—C7—H7C	109.5	C15—C19—H19C	109.5
H7A—C7—H7C	109.5	H19A—C19—H19C	109.5
H7B—C7—H7C	109.5	H19B—C19—H19C	109.5
C4—C8—H8A	109.5	C16—C20—H20A	109.5
C4—C8—H8B	109.5	C16—C20—H20B	109.5
H8A—C8—H8B	109.5	H20A—C20—H20B	109.5
C4—C8—H8C	109.5	C16—C20—H20C	109.5
H8A—C8—H8C	109.5	H20A—C20—H20C	109.5
H8B—C8—H8C	109.5	H20B—C20—H20C	109.5
C5—C9—H9A	109.5	C17—C21—H21A	109.5
C5—C9—H9B	109.5	C17—C21—H21B	109.5
H9A—C9—H9B	109.5	H21A—C21—H21B	109.5
C5—C9—H9C	109.5	C17—C21—H21C	109.5
H9A—C9—H9C	109.5	H21A—C21—H21C	109.5
H9B—C9—H9C	109.5	H21B—C21—H21C	109.5
Si1—C10—H10A	109.5	C18—C22—H22A	109.5
Si1—C10—H10B	109.5	C18—C22—H22B	109.5
H10A—C10—H10B	109.5	H22A—C22—H22B	109.5
Si1—C10—H10C	109.5	C18—C22—H22C	109.5
H10A—C10—H10C	109.5	H22A—C22—H22C	109.5
H10B—C10—H10C	109.5	H22B—C22—H22C	109.5
Si1—C11—H11A	109.5	C11—Si1—C10	105.92 (6)
Si1—C11—H11B	109.5	C11—Si1—C1	109.20 (6)
H11A—C11—H11B	109.5	C10—Si1—C1	109.90 (6)
Si1—C11—H11C	109.5	C11—Si1—Si2	110.88 (4)
H11A—C11—H11C	109.5	C10—Si1—Si2	110.06 (5)
H11B—C11—H11C	109.5	C1—Si1—Si2	110.77 (4)
Si2—C12—H12A	109.5	C12—Si2—C13	106.44 (6)
Si2—C12—H12B	109.5	C12—Si2—C14	109.04 (6)
H12A—C12—H12B	109.5	C13—Si2—C14	108.71 (6)
Si2—C12—H12C	109.5	C12—Si2—Si1	111.24 (4)
H12A—C12—H12C	109.5	C13—Si2—Si1	111.25 (4)
H12B—C12—H12C	109.5	C14—Si2—Si1	110.05 (4)
			
C5—C1—C2—C3	4.99 (13)	C20—C16—C17—C21	−0.01 (19)
Si1—C1—C2—C3	−114.20 (10)	C16—C17—C18—C22	177.49 (12)
C5—C1—C2—C6	−174.52 (12)	C21—C17—C18—C22	−0.1 (2)
Si1—C1—C2—C6	66.29 (14)	C16—C17—C18—C14	−2.63 (14)
C1—C2—C3—C4	−3.13 (14)	C21—C17—C18—C14	179.77 (12)
C6—C2—C3—C4	176.37 (12)	C15—C14—C18—C17	3.78 (13)
C1—C2—C3—C7	177.06 (12)	Si2—C14—C18—C17	−119.32 (10)
C6—C2—C3—C7	−3.4 (2)	C15—C14—C18—C22	−176.33 (11)
C2—C3—C4—C5	−0.26 (15)	Si2—C14—C18—C22	60.57 (14)
C7—C3—C4—C5	179.56 (12)	C5—C1—Si1—C11	−179.05 (8)
C2—C3—C4—C8	−179.61 (12)	C2—C1—Si1—C11	−64.46 (10)
C7—C3—C4—C8	0.2 (2)	C5—C1—Si1—C10	65.17 (10)
C3—C4—C5—C1	3.55 (14)	C2—C1—Si1—C10	179.76 (9)
C8—C4—C5—C1	−177.13 (12)	C5—C1—Si1—Si2	−56.66 (9)
C3—C4—C5—C9	−176.51 (12)	C2—C1—Si1—Si2	57.93 (9)
C8—C4—C5—C9	2.8 (2)	C15—C14—Si2—C12	61.62 (10)
C2—C1—C5—C4	−5.18 (13)	C18—C14—Si2—C12	178.86 (9)
Si1—C1—C5—C4	114.56 (10)	C15—C14—Si2—C13	177.27 (9)
C2—C1—C5—C9	174.88 (12)	C18—C14—Si2—C13	−65.49 (10)
Si1—C1—C5—C9	−65.38 (14)	C15—C14—Si2—Si1	−60.65 (9)
C18—C14—C15—C16	−3.64 (13)	C18—C14—Si2—Si1	56.59 (9)
Si2—C14—C15—C16	119.27 (10)	C11—Si1—Si2—C12	−178.64 (6)
C18—C14—C15—C19	175.62 (11)	C10—Si1—Si2—C12	−61.77 (7)
Si2—C14—C15—C19	−61.48 (14)	C1—Si1—Si2—C12	59.96 (6)
C19—C15—C16—C17	−176.98 (12)	C11—Si1—Si2—C13	62.88 (7)
C14—C15—C16—C17	2.25 (14)	C10—Si1—Si2—C13	179.74 (6)
C19—C15—C16—C20	0.9 (2)	C1—Si1—Si2—C13	−58.52 (6)
C14—C15—C16—C20	−179.88 (12)	C11—Si1—Si2—C14	−57.68 (6)
C15—C16—C17—C18	0.24 (14)	C10—Si1—Si2—C14	59.19 (6)
C20—C16—C17—C18	−177.76 (12)	C1—Si1—Si2—C14	−179.08 (5)
C15—C16—C17—C21	177.99 (12)		

### Hydrogen-bond geometry (Å, º)

Cg1 is the centroid of the C15–C18 ring.

**Table:**

*D*—H···*A*	*D*—H	H···*A*	*D*···*A*	*D*—H···*A*
C1—H1···*Cg*1^i^	1.00	2.65	3.646	173

Symmetry code: (i) −*x*+3/2, *y*−1/2, −*z*+1/2.
