# Supplementary figures and images for: Crystal structure of 1,1,2,2-tetra­methyl-1,2-bis­(2,3,4,5-tetra­methyl­cyclo­penta-2,4-dien-1-yl)disilane
Source: Acta Crystallogr E Crystallogr Commun. 2015 Oct 28;71(Pt 11):o888. doi: 10.1107/S2056989015019891 (PMC4644997; doi:10.1107/S2056989015019891)

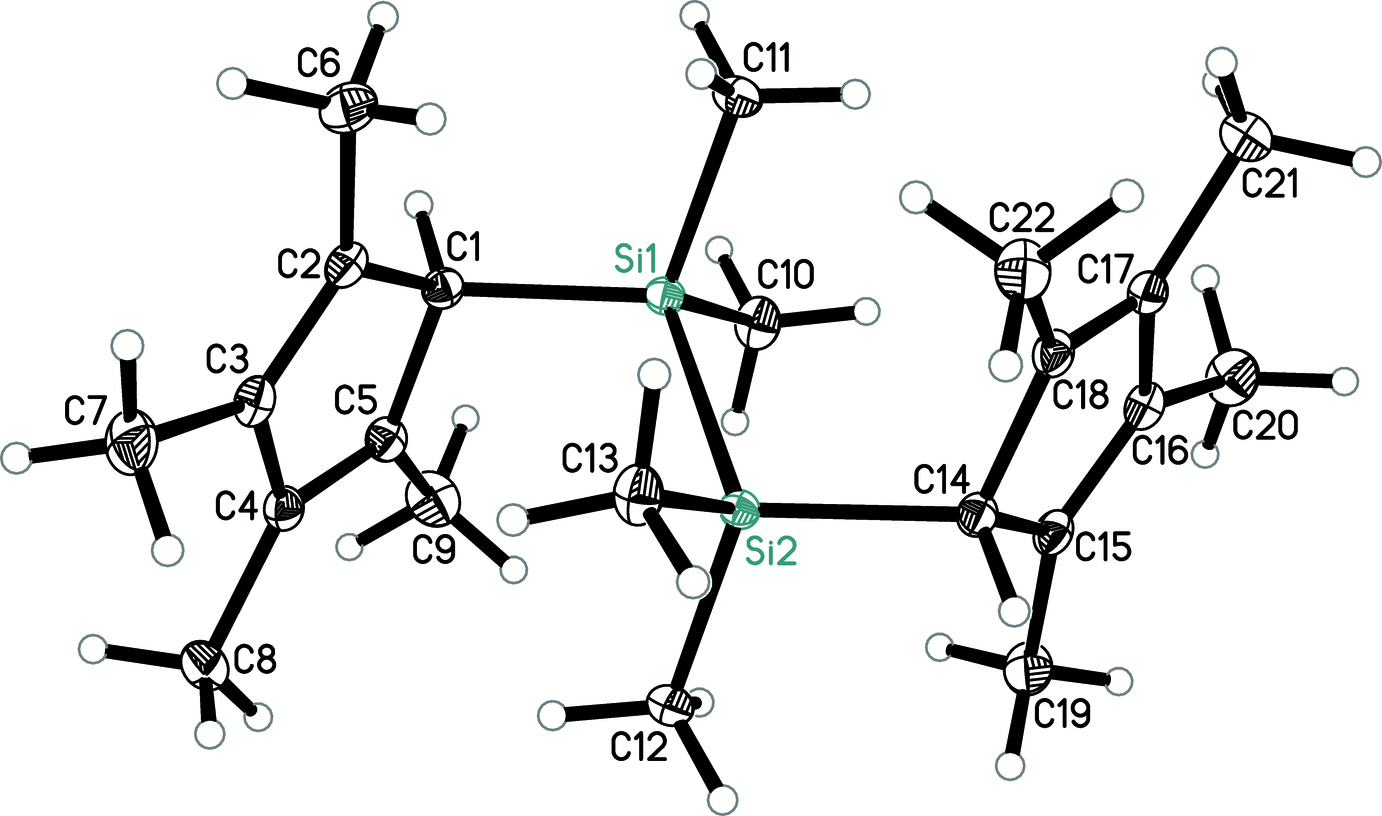

Supplement: Supplementary file 4 [file e-71-0o888-fig1.tif]
